# Course of post-traumatic stress disorder and health care utilisation among resettled refugees in the Netherlands

**DOI:** 10.1186/1471-244X-14-90

**Published:** 2014-03-27

**Authors:** Majda Lamkaddem, Karien Stronks, Walter D Devillé, Miranda Olff, Annette AM Gerritsen, Marie-Louise Essink-Bot

**Affiliations:** 1 Department of Public Health, Faculty of Medicine, University of Amsterdam, Amsterdam, the Netherlands; 2 Netherlands Institute for Health Services Research (NIVEL), Utrecht, the Netherlands; 3 Medical Anthropology and Sociology Unit, Faculty of Social and Behavioural Sciences, University of Amsterdam, Amsterdam, the Netherlands; 4 Pharos, Utrecht, the Netherlands; 5 Center for Psychological Trauma, Academic Medical Center, University of Amsterdam, Amsterdam, the Netherlands & Arq Psychotrauma Expert Group, Diemen, the Netherlands; 6 Epi Results, Louis Trichardt, South Africa

## Abstract

**Background:**

Post-traumatic stress disorder (PTSD) is a major health problem among refugees worldwide. After resettlement, the prevalence of PTSD remains high despite the fact that various PTSD treatments are known to be effective.

**Methods:**

We examined the course of PTSD and the role of mental health care utilisation at a 7-year interval (2003–2010) among a cohort of refugees from Iran, Afghanistan, and Somalia after resettlement in the Netherlands.

**Results:**

The unchanged high prevalence of PTSD (16.3% in 2003 and 15.2% in 2010) was attributable in part to late onset of PTSD symptoms (half of the respondents with PTSD at T2 were new cases for whom PTSD developed after 2003). The second reason concerned the low use of mental health care services at T1. Whereas the multivariate analyses showed the effectiveness of mental health care, only 21% of respondents with PTSD at T1 had had contact with a mental health care provider at that time. Use of mental health care during the first wave increased the odds of improvement in PTSD symptoms between both measurements (OR 7.58, 95% CI 1.01; 56.85).

**Conclusions:**

The findings of this study suggest there are two possible explanations for the persistently high prevalence of PTSD among refugees. One is the late onset of PTSD and the other is the low utilisation of mental health care. Health care professionals should be aware of these issues, especially given the effectiveness of mental health care for this condition.

## Background

Post-traumatic stress disorder (PTSD) is a major health problem among refugees worldwide [1]. A review by Fazel and colleagues [2] showed that the overall prevalence of PTSD among refugees resettled in Western countries was about 9%, with substantial heterogeneity between studies. Although this overall percentage was not as high as that suggested by others [3-5], Fazel and colleagues suggested that refugees resettled in Western countries are about ten times more likely to have PTSD than age-matched general populations in those countries. For example, in the Netherlands, the lifetime prevalence of PTSD is 7.4% for the general population [6], whereas this is around 20% for refugees and asylum seekers from Iran, Afghanistan, and Somalia [5].

While the proportion of refugees with PTSD is smaller after resettlement in the host countries, it remains relatively high compared with the general population [5,7]. Although this might indicate the chronicity of a severe mental illness, this persistently high prevalence seems to be at odds with the availability of effective forms of treatment for PTSD [8,9]. Since several PTSD treatments are known to be effective among diverse groups, the question arises as to why there is little change in the proportion of resettled refugees with PTSD, even several years after resettlement [5].

A first possibility, or hypothesis, is that although the prevalence of PTSD remains high, it represents PTSD in different subjects. An almost unchanged proportion of refugees with PTSD over time does not necessarily mean that the positive cases are in fact the same persons. Late onset of PTSD several years after the traumatic events took place is highly probable [10], and this must be taken into account when examining changes over time. Late-onset PTSD has been shown up to 14 years after the traumatic events [11].

A second hypothesis is that the available treatment services are inadequately used by refugees with PTSD, implying underutilisation. Timely use of appropriate mental health care is considered necessary for recovery from PTSD. A prompt intervention based on cognitive behavioural treatment can relieve the complaints and prevent the development of PTSD [12-14]. Dutch guidelines for general practitioners (GPs) recommend direct referral to mental health care (Dutch College of General Practitioners’ Standard M62) [15]. The regulations for access to Dutch health care provide refugees, asylum seekers, and Dutch citizens with similar rights to primary health care and specialised mental health care through GP referrals. However, a distrust of mental health care, a lack of knowledge about mental health treatment possibilities, and language barriers might limit access to mental health care among this group of newcomers, which is unfamiliar with the new country’s health system. A study conducted in the United States showed that, in the years following resettlement, many refugees do not receive adequate care [16]. Language barriers, acculturation issues, and cultural beliefs about several forms of health care contribute jointly to an access problem for this group.

Finally, a third hypothesis is that mental health care may not have had the expected positive effect on the course of mental health for refugees who used mental health care services. For refugees, the ineffectiveness of mental health care treatments for PTSD might account for long-lasting symptoms. Although current guidelines recommend trauma-focused psychotherapy for patients with PTSD [17,18] and effective forms of treatment for PTSD are available [8,9], very limited or no effectiveness has been reported for PTSD treatment specifically for asylum seekers and refugees [19,20]. Although two pilot studies demonstrated the feasibility and effectiveness of some trauma-focused approaches for treating PTSD in refugees [21,22], more evidence of treatment effectiveness is needed for this group.

The second and third hypotheses concern the use and effectiveness of mental health care. The role of mental health care utilisation in the course of mental health is embedded in the resettlement situation of refugees, and is also related to pre-resettlement events. Traumatic events preceding the flight, whether experienced or witnessed, are direct risk factors for the onset of mental illness [23], as well as for its persistence [7,24]. Post-migration factors can also jeopardise mental health [25]. Among post-migration factors, living difficulties related to employment, social and family networks, dealing with a new culture, and social position show a direct relationship to mental illness among resettled refugees [Lamkaddem et al., submitted]. Therefore, these pre- and post-migration factors must be taken into account when examining the role of mental health care utilisation in the course of PTSD.

A longitudinal study design was required to answer our research question on how to explain the persistently high prevalence of PTSD among resettled refugees. Therefore, we examined the course of PTSD and the related role of mental health care utilisation among a cohort of refugees from Iran, Afghanistan, and Somalia shortly after resettlement in the Netherlands.

## Methods

### Study population

In 2003–2004, a sample of 410 refugees from Iran, Afghanistan, and Somalia both with and without residence permits (178 asylum seekers and 232 residence permit holders) was interviewed for the baseline measurement (T1). A more detailed description of the initial study sample and measurements can be found elsewhere [26].

**Figure 1:**
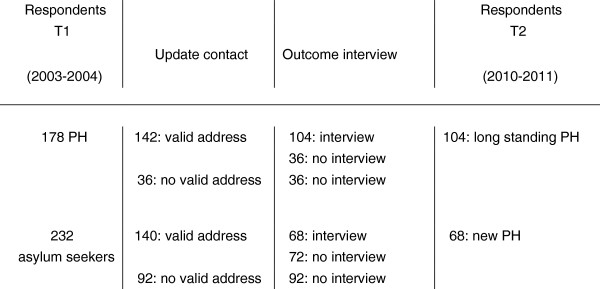
**Overview of data collection.** PH = residence permit holders.

In 2010–2011, respondents were invited by mail to participate in the second wave (T2). This period of 7 years between both waves was dictated by practical and financial circumstances, and also by the fact that there had to be enough time between both waves to allow a) new residence permit holders to resettle and b) longstanding residence permit holders to experience changes in health state. Of the 410 T1 participants, 128 had no known address in the Netherlands because they had not been granted asylum (n = 65), because the address we had was not valid (incorrect or outdated) (n = 59), or because they had died (n = 4). This left a sample of 282 refugees, most of whom had been granted residence permits since the first wave (Figure 1). Of these 282 refugees, 172 were interviewed for follow-up (response rate: 61%, retention rate: 42%). Most of those who did not respond (n = 110) could not be reached (43% of non-response) or refused to participate (30% of non-response).

At both T1 and T2, questionnaire-based face-to-face interviews were conducted in the participants’ language of choice (Dutch, Dari, Pashto, Farsi, or Somali). Interviewers and respondents were matched on gender and ethnic background. Written informed consent (translated, if necessary) was obtained from all respondents. According to Dutch law (the Medical Research Involving Human Subjects Act), formal ethical approval was not required. However, the study protocol for the baseline measurement was approved by the Medical Ethics Committee of the VU University Medical Center in Amsterdam [26], and we took every precaution to guarantee the respondents’ anonymity.

### Socio-demographic variables

Information on age and gender was available from the survey and registers used for sample selection at T1 (for more details on the registers, see Gerritsen et al. [26]).

#### *Pre-migration traumatic events*

Traumatic experiences preceding the flight were assessed with Part I of the Harvard Trauma Questionnaire (HTQ), which includes 17 events (e.g. lack of food and water; being close to death) [27]. Events experienced and/or witnessed were coded as 1 (vs 0 = ‘no/heard about’). The total number of events experienced and/or witnessed (i.e. the sum of all items for which the response differed from ‘no’) was subsequently computed. For more details on the HTQ items and the motivation for coding answer categories, see the paper by Gerritsen et al. on the study design of the first wave of the study [26].

### PTSD

PTSD was measured using Part IV of the HTQ [27]. For the present study, only the first 16 items of the 30 items on the HTQ were taken into account; the remaining 14 items were considered less specific for PTSD [26]. Individuals with a mean score of ≥ 2.5 (range: 1–4) on the first 16 items were considered to have PTSD. This cut-off point is widely used in studies on refugees, and was therefore chosen for purposes of comparability (Gerritsen et al. [26]). Change scores were determined by subtracting scores at T1 from scores at T2. Scores < 0 were considered as a deterioration, scores > 0 as an improvement. Scores = 0 were coded as ‘no change’. For the multivariate analyses, this information was dichotomised as 1 = improvement in PTSD score between T1 and T2, and 0 = deterioration in PTSD score between T1 and T2. The ‘no change’ option rarely occurred at individual level; such cases were left out of the multivariate models.

### Mental health care use in the Netherlands

At T1, respondents were asked whether they had had any contact with a mental health care provider in the past 12 months. The options included social workers, psychologists, psychiatrists, psychotherapists, and other (unspecified) kinds of psychosocial care providers. For use in the multivariate models, the answers were dichotomised into 0 (‘no contact with any of these mental health care providers’) and 1 (‘contact with at least one of these mental health care providers’).

### Post-migration living difficulties

The respondents were asked about possible stressful experiences they had experienced in the Netherlands. The checklist included 18 problems often reported by refugees in research on post-migratory stressors (e.g. delays in the application for a residence permit; loneliness) [28-30]. Respondents were asked to indicate the extent to which any of these problems had bothered them in the previous month (1 = ‘not at all’ to 4 = ‘extremely’). All items together were considered to form a scale as shown in previous studies [29,31]. A mean score was calculated (range: 1–4) for both T1 and T2. Change scores were calculated by subtracting scores at T1 from scores at T2.

### Analyses

We first examined the course of symptoms of PTSD at a 7-year interval (first hypothesis on the late onset of PTSD). Second, we examined the extent to which refugees with PTSD during the first and second waves reported mental health care utilisation at that time (second hypothesis on mental health care utilisation). Finally, we examined the course of the symptoms in relation to prior mental health care utilisation (third hypothesis on effectiveness of mental health care).

#### *Course of PTSD between T1 and T2*

To examine the possible late onset or persistence of PTSD, respondents with PTSD at T2 but not at T1 were shown as a percentage of the total study sample. Similarly, the proportion of respondents with persistent PTSD was calculated as a percentage of the total number of respondents with PTSD at T1. The change in PTSD symptom severity was further examined by looking at the average scores at T1 and T2, using paired samples t-tests.

#### *Mental health care utilisation at T1*

To examine the use of health care in relation to PTSD, cross tables and chi-square tests were used to present and test the association between having PTSD at T1 or T2 and using mental health care (yes/no) simultaneously (yes/no).

#### *Improvement in PTSD symptoms between T1 and T2, and pre- and post-migration factors and mental health care utilisation*

Pre- and post-migration factors were taken into account when examining the effectiveness of mental health care use on PTSD symptom severity. To do so, a logistic regression model was used to assess the multivariate association of improvement in PTSD score between T1 and T2 (dependent variable) with the number of traumatic events witnessed/experienced before the flight (measured at T1), the change in number of experienced post-migration living difficulties between T1 and T2, and the mental health care utilisation in the 12 months prior to T1. The analyses were adjusted for age, gender, mental health care utilisation at T2, and PTSD score at T1. All analyses were performed using SPSS 16.00 for Windows.

## Results

### Study population

Table 1 presents the main socio-demographic characteristics of the initial study sample and those of participants in both waves. Proportionally, participants in both T1 and T2 did not differ significantly from the initial study sample regarding the main socio-demographic characteristics (p > 0.05), except for residence permit and country of origin (p < = 0.05). The two-wave cohort included proportionally more permit holders than asylum seekers (status at T1). This resulted from a selection of the initial sample: not all asylum seekers at T1 had obtained residence permits at T2 (see Methods), which explains the over-representation of longstanding residence permit holders in the two-wave cohort.

**Table 1:** Socio-demographic characteristics of participants in one (T1) and both waves (T1 and T2)

	**T1 (n = 410)**	**T1 and T2 (n = 172)**
Country of origin		
Afghanistan	206 (50.2)	82 (47.7)
Iran	117 (28.5)	63 (36.6)
Somalia	87 (21.2)	27 (15.7)
Age in years: mean at T1 (SD)	37.0 (12.4)	39.1 (13.1)
Residence status		
Permit holder at T1 (= longstanding permit holder at T2)	178 (43.4)	104 (60.5)
Asylum seeker at T1 (= new permit holder at T2)	232 (56.6)	68 (39.5)
Gender		
Male	241 (58.8)	84 (48.8)
Female	169 (41.2)	88 (51.2)
Education at T1	*(T1: n = 408)*	
None/primary	109 (26.6)	35 (20.7)
Secondary	142 (34.6)	62 (36.0)
Vocational/University	159 (38.8)	75 (43.3)
Marital status at T1	*(T1: n = 408)*	
Divorced	17 (4.2)	7 (4.1)
Never married	40 (9.8)	20 (11.6)
Married/living together	252 (61.6)	111 (64.5)
Widowed	100 (24.4)	34 (19.8)

### Course of PTSD

**Figure 2:**
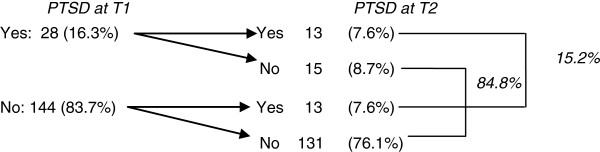
Number of respondents who scored above the PTSD cut-off point at T1 and T2.

Figure 2 shows the number of respondents with PTSD during the first and second waves, using the standard cut-off point of ≥ 2.5 (see Methods). During the first wave, 16.3% (n = 28) of the respondents had scores above the cut-off point, compared with 15% (n = 26) during the second wave.

When using paired measurement methods to examine changes in average PTSD scores from Part IV of the HTQ, the average at T1 (1.81; standard deviation (SD): 0.68) and at T2 (1.74; SD: 0.69) showed no significant difference (t = 1.315; p = 0.190). Thus, based on the severity of experienced symptoms, respondents showed (on average) no improvement at T2 compared with their mean score at T1. However, when examining the time of onset of PTSD, 50% of those with PTSD at T2 already had PTSD at T1 (n = 13), while the remaining 50% were new cases (i.e. they did not have PTSD at T1) (n = 13). Also, 53.6% of the respondents with PTSD at T1 recovered (46.4% remained symptomatic).

### Mental health care utilisation and PTSD at T1

At T1, examination of the association between PTSD symptom severity and use of mental health care showed that those reporting having used mental health care also reported significantly higher scores of PTSD at T1 (2.42 vs 1.75, t = -3.204, p = 0.006). Moreover, Table 2 shows that, at T1, most respondents who had PTSD (≥ the cut-off point of 2.5) did not use mental health care at T1. Only 21% of respondents with PTSD had contact with a mental health care provider at that time, while 79% did not (chi square = 6.785, p = 0.009). At T2, the proportion of respondents with PTSD who used mental health care at T2 increased to 54% (Table 2). Also, for the group below the PTSD cut-off point at T2, the proportion of respondents who used mental health care doubled compared with the situation at T1 (13% vs 6%).

**Table 2:** Respondents above PTSD cut-off point and use of mental health care at T1 and T2

		**Contact with mental health care provider at T1 or T2**		
		**No contact**	**Contact**	**Total**
PTSD	At T1	22 (78.6%)	6 (21.4%)	28 (16.3%)
	At T2	12 (46.2%)	14 (53.8%)	26 (15.2%)

### Improvement in PTSD symptoms, pre- and post-migration factors, and mental health care utilisation

When migration and socio-demographic factors were taken into account, prior mental health care use (during the first wave) was significantly associated with an improvement in PTSD score between both waves (Table 3). In other words, regardless of differences in the above-mentioned covariates, those who made use of mental health care during the first wave were up to seven times more likely to see an improvement in their PTSD symptoms than those who did not. However, due to the small numbers, these data do not provide an accurate estimate. The large confidence interval (CI) indicates that caution should be used when interpreting these results.

**Table 3:** **Logistic regression coefficients of improvement in PTSD score between T1 and T2**^**1**^

	**OR (95% CI)**	**p-value**
Contact with mental health care provider at T1 (ref: no contact)	7.59 (1.01; 56.86)	0.049
Change in living difficulties between T1 and T2	0.81 (0.71; 0.94)	0.004
Number of traumatic events	0.90 (0.79; 1.03)	0.130

^1^Adjusted for age, gender, contact with mental health care provider at T2, and PTSD score at T1.

Furthermore, the number of pre-migration traumatic events was not significantly associated with an improvement in PTSD score (odds ratio (OR) = 0.905, p > 0.05). An increase in experienced post-migration living difficulties between T1 and T2 was negatively related to an improvement in PTSD score between both waves (OR = 0.813, p < 0.05).

## Discussion

In this paper, we examined the course of PTSD among refugees after resettlement. The results show that there are two main explanations for the seemingly unchanged high rate of refugees with PTSD at a 7-year interval.

The first explanation concerns the onset and persistence of PTSD symptoms. Only half of the respondents who had PTSD during the second measurement also had PTSD during the first measurement. The other half of the respondents were new cases who developed PTSD later (i.e. between the two measurements).

The second explanation concerns the use of mental health care at T1. We saw that at T1, relatively few respondents with PTSD had had contact with a mental health provider (21%). We were more likely to see an improvement in PTSD symptoms during the second wave for those who had made use of mental health care during the first wave, which suggests that the low use of health care services also contributes to the remaining high prevalence of PTSD.

Our results on the course (onset and persistence) of PTSD are confirmed by other longitudinal studies among different ethnic groups (whether refugees or not). PTSD symptoms can persist several years after exposure to trauma [32,33], and can also have a delayed onset, whereby symptoms only become apparent several years after the traumatic events have taken place [34,10]. These results stress the importance of a longitudinal cohort design when examining the course of PTSD. Our study shows that late onset of PTSD is an important reason for the ongoing high prevalence of PTSD after resettlement.

Our results on the effectiveness of mental health care provide evidence for the effect of mental health care utilisation for this group. In refugees who had used mental health care, PTSD symptoms generally improved. These findings are confirmed by the few studies that specifically addressed this topic among refugees. One Dutch clinical study reported that 73% of patients diagnosed with PTSD no longer met the criteria for the diagnosis 6 months after treatment, whereas 90% of those who had refused treatment were later diagnosed with PTSD [35]. Therefore, for refugees, accessing and using mental health care is an important precondition for improving PTSD status.

While mental health care seems to be beneficial to PTSD recovery, our results show that the percentage of refugees who actually use mental health care services is relatively low (i.e. one fifth of those reporting PTSD symptoms). Although these results are similar to studies in other countries of resettlement, few have specifically examined Dutch mental health care services. The last study on this topic was conducted among Iraqi asylum seekers [36], and indicated a large unmet need for mental health care: over 90% of asylum seekers with a psychiatric disorder did not visit a Dutch mental health care service. In the present study among refugees with PTSD, this percentage was almost 80%. The reasons for the low use of mental health care services among refugees need to be investigated further. Barriers to accessing mental health care can be due to low language fluency [37], lack of knowledge on existing treatments [38], and culturally and/or linguistically unsuitable forms of information about the mental health care supply. Just as for other types of migrants [39], these factors might in turn affect the perceived mental health care needs of refugees, and impede access.

The present study has several limitations. First, only 172 respondents took part in both waves of the study. This might affect analyses on the extent to which pre-migration factors (i.e. traumatic events before the flight) impact the course of PTSD after resettlement. The same analyses among a larger sample might show a significant (negative) association between the number/severity of traumatic events and PTSD recovery. Also, it was not possible to accurately assess the extent to which those who made use of mental health care during the first wave were more likely to see an improvement in their PTSD symptoms (the large 95% CI reflects the uncertainty of the estimation).

Second, the relatively high attrition rate between the first and second wave might have induced some bias. However, non-inclusion was mainly attributable to remigration (30% of the initial sample), which was unavoidable in these types of settings. The non-response analysis presented in Table 1 shows that the socio-demographic characteristics of our study sample do not differ from the initial sample. Moreover, given the objective of our research and the focus on the explanatory factors rather than on the prevalence of PTSD, we do not expect the attrition to have had a considerable impact on our results.

Third, our study included three different ethnic groups, which represent only part of the refugee population in the Netherlands. The extent to which these results can be generalised to all refugee groups in the Netherlands and elsewhere remains to be confirmed in larger research samples.

Fourth, the measurement of PTSD is not a clinical one, and the questionnaire used for this purpose must be seen as only an approximation of PTSD. However, studies have successfully used the HTQ (or parts of it), yielding results comparable to clinical diagnoses [40,41].

Finally, the information on the use of mental health care services was not directly linked to the diagnosis. We have no evidence that the reported contacts with mental health care providers reported by refugees with PTSD symptoms were in fact specifically for PTSD treatment. Therefore, we can draw no conclusions on the effectiveness of a specific PTSD treatment, but merely on the association between the use of mental health care in general and the course of PTSD symptoms.

## Conclusions

Despite these limitations, the explanations presented here for the seemingly unchanged high rates of PTSD offer interesting insights for further research on the mental health of refugees. The findings emphasise the need for primary care providers to follow existing guidelines on quick referral to mental health care for patients presenting with PTSD symptoms, and underline the possibility of late-onset PTSD. Finally, in addition to the use of mental health care, the results show the importance of improvements in contextual factors (e.g. in employment, social/family networks, becoming familiar with the new culture, and social position) on the course of PTSD.

## Competing interests

The authors declare that they have no competing interests.

## Authors’ contributions

ML carried out the study, performed the statistical analyses, and drafted the manuscript. MLE and KS participated in the design and coordination of the study, and helped draft the manuscript. AG, WD, and MO contributed to drafting the manuscript. All authors read and approved the final manuscript.

## Pre-publication history

The pre-publication history for this paper can be accessed here:

http://www.biomedcentral.com/1471-244X/14/90/prepub
